# Characterization of type-specific HPV prevalence in a population of persistent cutaneous warts in Flanders, Belgium

**DOI:** 10.1038/s41598-023-44154-y

**Published:** 2023-10-15

**Authors:** Nina Redzic, A. Rita Pereira, Sonia Menon, Johannes Bogers, Astrid Coppens, Kaat Kehoe, Davy Vanden Broeck

**Affiliations:** 1 Laboratory of Molecular Diagnostics, AML - Sonic Healthcare Benelux, Antwerp, Belgium; 2 AMBIOR, Laboratory for Cell Biology and Histology, University of Antwerp, Antwerp, Belgium; 3 International Centre for Reproductive Health, Ghent University, Ghent, Belgium; 4 National Reference Centre for HPV, Brussels, Belgium; 5 Department of Obstetrics and Gynecology, Women’s Clinic, Ghent University Hospital, Ghent, Belgium

## Abstract

Cutaneous warts are benign skin lesions caused by the human papillomavirus (HPV). Even though they are considered benign, they can have a considerable impact on the quality of life and cause serious illness in certain immunocompromised populations. Studies have shown that the efficacy of wart treatment is dependent on the causative HPV type. Therefore, in this article, we aim to determine the HPV genotype-specific prevalence in cutaneous warts of a Flemish population as part of the Omnivirol-Salycilic acid randomized controlled trial. Swab samples of cutaneous warts (n = 269) were collected during enrollment. The DNA extraction was performed on the automated NucliSENS® easyMAG® system (bioMérieux). The samples were analyzed with two separate in-house PCR assays capable of detecting the most prevalent cutaneous HPV types (i.e. wart-associated HPV qPCR) as well as the most relevant mucosal types (i.e. RIATOL qPCR assay). In total, the type-specific prevalence of 30 distinct HPV genotypes was determined. The beta-globin gene was used as a cellularity control and for viral load quantification. Data concerning wart persistence, previous treatment, wart type, and other relevant wart and patient characteristics was collected through a baseline questionnaire. The study population consisted mostly of persistent warts considering that 98% (n = 263) of the sampled skin lesions were older than six months and 92% (n = 247) had undergone previous treatment. The most prominent wart type was the mosaic verruca plantaris (42%, n = 113). The most prevalent HPV types were cutaneous HPV types 27 (73%, n = 195), 57 (63%, n = 169), and 2 (42%, n = 113). Only 2% (n = 6) of the lesions was HPV negative. The highest median viral loads were observed with HPV27 and 57 (i.e. 6.29E+04 and 7.47E+01 viral copies per cell respectively). The multivariate analysis found significant associations between wart persistence and certain wart types, the number of warts, and HPV genotypes. Based on these findings, persistent warts are more likely to: (1) be verruca vulgaris, verruca plantaris simple or mosaic, (2) to manifest as multiple warts, (3) and to be negative for HPV type 2 or 4. These characteristics can be useful in the clinical setting for future risk stratification when considering treatment triage and management.

*Trial registration*: NCT05862441, 17/05/2023 (retrospectively registered).

## Introduction

Cutaneous warts are benign lesions of the skin, commonly found and widespread among the population, affecting both children (30%) and adults (0.84%-12.9%), with a typically long duration of disease (approx. two years)^1,2^. Clinical presentations of warts show a wide variety, including dome-shaped keratotic lesions with exophytic growth, endophytic keratotic papules, and flat-topped papules^3,4^. Depending on their appearance and location, warts are categorized into different types, comprising common warts (verrucae vulgaris), plantar warts (verrucae plantaris simple and mosaic), filiform warts (verrucae filiformis) and plane warts (verrucae plana)^5^. Warts can be found highly prevalent among the population, enriched in children and immunocompromised patients^6–8^. Roughly one-third of schoolchildren have warts, of which the majority clear spontaneously within two years^8,9^. Due to the discomfort they cause, patients often present themselves for treatment rather than waiting for spontaneous clearance^10^. Common first-line treatments sought out by affected individuals, include folk remedies (e.g. garlic, thuja tincture, taping) and over-the-counter acids in low concentrations (e.g. salicylic acid, lactic acid, acetic acid). These treatments always require long-term application, often with unsatisfactory results, which is why patients frequently resort to second-line treatment administered by a healthcare professional, including cryotherapy, higher concentrations of salicylic acid, excision or locally applied immunomodulatory or antimitotic drugs (e.g. imiquimod, bleomycin, fluorouracil)^11–15^. Despite long-term, intensive treatment schemes, patients are often confronted with the recurrence of warts, inducing frustration and mental fatigue^16^.

A systematic review by the Cochrane Skin Group assessed the effects of different treatments for cutaneous warts. It concluded that the rate of clearance remains highly uncertain even after treatment, hereby influencing practitioners to limit treatment efforts and instead opt for a more expectant approach^17^. In concordance with this systematic review, Kuwabara et al. found that 80% of warts do resolve spontaneously within 4 years, regardless if they have been treated or not^18^. However, this still means that the remaining 20% of warts are strongly persistent. Currently, there is no reliable mean of predicting which warts will clear spontaneously and which will remain persistent for years.

Cutaneous warts are caused by infection of the skin with human papillomavirus (HPV), mostly known as the cancer-causing virus, capable of inducing cervical cancer^19^. The HPV family comprises over 200 genotypes, classified into different types according to their DNA sequence^20^. The most prevalent HPV types found in warts are HPV 1, 2, 3, 4, 7, 10, 27, 41, 57, 60, 63 and 65^3,21–24^. A noteworthy study by Bruggink et al. explored the presence of selected HPV genotypes in relation to natural course and treatment response and provided early insights in the causal relationship between HPV genotype and treatment efficacy/lesion resolution^25^. However, the authors were not able to formulate statements about most of the tested HPV types, due to absence of sufficient numbers per HPV type in different treatment groups. Other studies have equally suggested that the efficacy of a wart treatment could be dependent on the causative HPV type, hence indicating a role for HPV genotyping in clinical management and therapy selection^26,27^. Combining insights from these diverse explorations, a single-center randomized controlled trial, i.e. Omnivirol-Salicylic acid combination therapy for cutaneous warts (OVW-SA), was designed to explore further the predictive value of HPV genotyping in relation to treatment efficacy and time to clearance^28^.

This article aims to present baseline findings of the study population included in the OVW-SA trial conducted in Flanders, Belgium, comprising complex inter-relationships between patient-specific features as well as wart-specific characteristics, including the most extensive HPV genotyping performed on wart swab samples to date. Based on these findings a detailed epidemiological profile of a typical persistent wart is devised.

## Methods

### Setting and population

Patients were included as part of the OVW-SA001 clinical trial conducted in a Belgian population between 2018 and 2019^29^. The study consisted of a double-blind, single-centered, randomized clinical trial that was conducted by the University of Antwerp, with Sonic Healthcare BeNeLux (Antwerp, Belgium) operating as the central laboratory. Ethical approval was obtained from the Ethical Review Board of the University of Antwerp (B300201734040). The study was conducted in accordance with applicable national regulations, Good Clinical Practice (2005/28/EC)^30^ and the Declaration of Helsinki^31^. Informed consent was obtained from all participants prior to study entry. In case of minors, informed consent was obtained from legal guardians before study participation. Recruitment was done via flyers distributed via dermatologists, pharmacists and via own initiatives aiming to reach an immunocompetent population exhibiting one or more cutaneous warts, aged 12 years or older, and agreeing to sign informed consent written in Dutch. The study area comprised the Flemish region with enrichment in the Antwerp metropolitan area.

### Study design

In total 269 patients were included, fulfilling inclusion criteria, hereby reaching pre-calculated statistical power. At inclusion, data concerning wart duration, previous treatment, wart type and other relevant wart and patient characteristics were collected through a structured baseline questionnaire (Additional File 1). Full details of clinical trial design and methodology are provided in Redzic et al.^28^.

### Study procedures

At inclusion, a photograph of the index wart (i.e. the largest wart) was taken, followed by sampling with a Floq swab (FLOQSwab Copan Diagnostics, Murietta, California, USA). Sample-taking procedures and processing details were done as described by Redzic et al.^32^. The sample was stored in Abbott multi-Collect medium (Multi-Collect Specimen Collection Kit, Abbott Molecular Inc., Des Plaines, Illinois, USA) and kept at 4 °C prior to further processing. Briefly, DNA extraction was performed according to the optimized method for cutaneous samples on the automated NucliSens easyMAG platform (bioMérieux, Boxtel, The Netherlands). The samples were analyzed for the presence of HPV DNA by the in-house developed wart-associated cutaneous HPV assay, capable of detecting the above-mentioned 12 distinct cutaneous HPV types (i.e. HPV 1, 2, 3, 4, 7, 10, 27, 41, 57, 60, 63, 65)^33^, as well as the RIATOL HPV genotyping qPCR assay, capable of detecting mucosal HPV types 6, 11, 16, 18, 31, 33, 35, 39, 45, 50, 51, 52, 53, 56, 59, 66, 67 and 68^34^. The RIATOL qPCR assay is a fully validated HPV test, used to conduct primary HPV screening for cervical cancer^35,36^. This study used the assay without clinical cut-off at its full analytical potential. Both assays allow for quantitative measurement of viral load after calibration with synthetic gBlock gene fragments (Integrated DNA Technologies, Coralville, Iowa, USA). Sample adequacy (cellularity control) was assessed via amplification of beta-globin, a housekeeping gene, which was similarly applied as a measure of the number of cells present essential for subsequent viral load calculations. In summary, all samples were both qualitatively as well as quantitively tested for the presence of 30 distinct HPV types. A detailed description of assay design and validation can equally be found in Redzic et al.^33^.

### Data management and analysis

Data management and analysis were conducted using STATA version 17 (Corporation, College Station, Texas, USA). In order to summarize data regarding patient- and wart-specific characteristics descriptive statistics such as numbers and percentages with 95% CIs were applied for categorical variables, and arithmetic mean, standard deviation, and median were used for numerical variables. The presence of any detectable viral load of a specific HPV type was considered diagnostic for infection with that specific type.

Kruskal–Wallis test was used to compare patients’ age, number of warts, number of multiple infections and estimated viral loads between different HPV types. Student T-test was used to assesses the number of warts between the two genders and differences in viral load between single and multiple infections. Results were considered statistically significant at *P*-value ≤ 0.05.

Potential predictors of wart persistence were assessed via a multiple logistic regression model, in which wart persistence was defined as index wart duration of more than 6 months combined with resistance to previous treatment. Several variables were created for analysis. Age was dichotomized into two categories: ≥ 13 years and < 12 years, as patients younger than 12 years are known to have higher rates of wart clearance^37,38^. The number of HPV infections was treated as a categorical variable, with no HPV as the baseline category, one HPV and two or more HPV multiple infections as other categories. The number of warts was categorized as 1 (baseline) versus 2 or more warts. The type of warts variable was divided into four categories, with verruca plantaris mosaic as the baseline, verruca plantaris simple, verruca vulgaris, and “others” (i.e. verruca plana and filiformis). For the univariate analysis, logistic regression was fitted to measure the strength of the association of potential covariates. For model building, all predictors that had a *P*-value of less than 0.2 were considered, in addition to the potential confounders hypothesized to be of importance: the type of wart, the number of warts, the number of infections and specific cutaneous HPV types that had more than 10 observations (i.e. HPV 1, 2, 3, 4, 10, 27, 57, 63, and 65).

As no other predictors were identified, a multivariable logistic regression analysis was performed to simultaneously control for potential confounders. For variables that were not dichotomized, interaction terms were fitted, and linear assumptions were tested. Both the Akaike information criterion (AIC) and the Bayesian Information Criterion (BIC) were used as two different measures of model fit, along with the Variance Inflation Factor (VIF), to detect multicollinearity to ensure that yielded values were within acceptable ranges^39^.

To obviate problems related to the stability of parameter estimates that arose for the variable HPV 63 due to the flatness of the likelihood method, the Penalization likelihood method was used instead of the standard Maximum likelihood method^40^.

### Ethics approval and consent to participate

Ethical approval obtained from ethics committee of Antwerp University Hospital, B300201734040. All participants gave written informed consent prior to entry into the study.

## Results

### Socio-demographics and wart-characteristics

**Figure 1:**
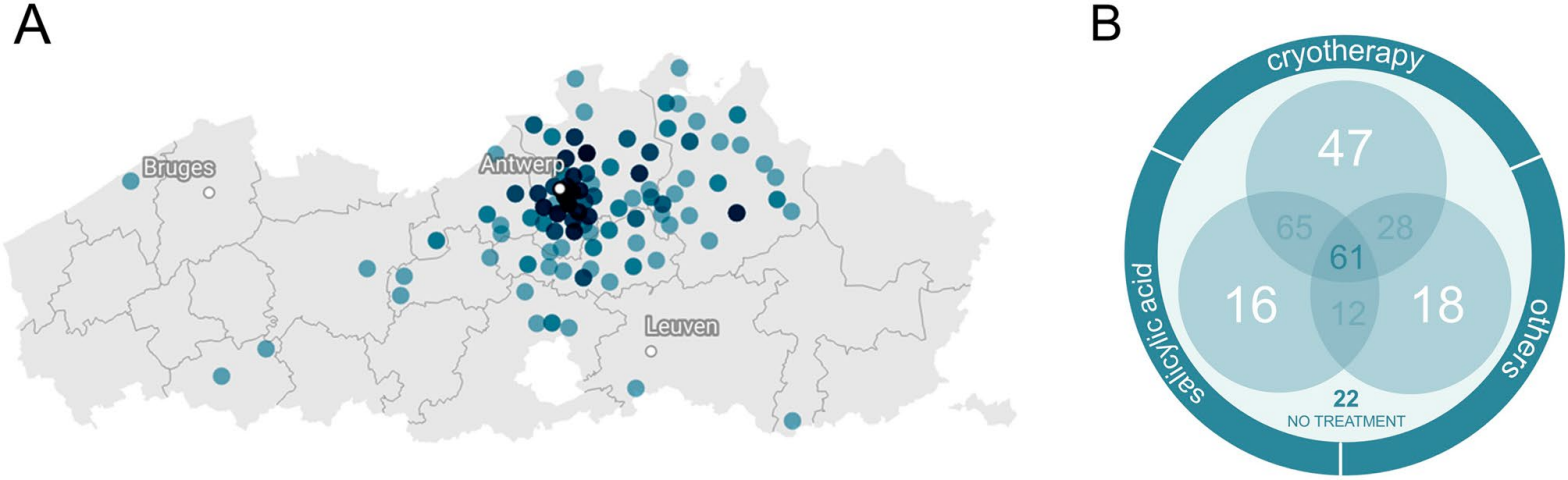
(**A**) Origin of samples collected during the OVW-SA001 clinical trial. Darker blue colors represent higher density of collections per geographical area. (**B**) Index wart treatment history. The Veen-diagram depicts the number of patients that applied different types of treatments on their index warts. In summary, 22 index warts did not have any previous treatment, while cryotherapy was the primary treatment for most index warts (n = 201), followed by salicylic acid (n = 154) and various other types of therapy (n = 119). The most common treatment combination was cryotherapy combined with salicylic acid (n = 65).

Figure 1A provides an overview of the geographical locations were all the study samples originated from, with most samples (89%) being collected from the Antwerp metropolitan region. A summary of the population- and wart-specific characteristics can be found in Table 1. Median age of participants was 39 (7–76) years for males and 38 (12–82) years for females, and 60.2% of the population was female. At study enrollment, 66 patients (24.5%) had one wart, 156 (58.0%) had 2–10 warts and 47 (17.5%) had more than 10 warts.

**Table 1:** Distribution of patient- and wart-specific characteristics among the study population.

Variable	Number of patients (n = 269)	Percentage (%)
Sex		
Male	107	39.8
Female	162	60.2
Age		
< 12 years	3	1.1
12–18 years	53	19.7
> 18 years	213	79.2
Total number of warts		
1	66	24.5
2–10	156	58.0
> 10	47	17.5
Index wart location		
Hands	85	31.6
Feet	176	65.4
Other	8	3.0
Index wart type		
Verruca vulgaris	89	33.1
Verruca plantaris mosaic	113	42.0
Verruca plantaris simple	60	22.3
Verruca plana	6	2.2
Verruca filiformis	1	0.4
Index wart duration		
< 6 months	6	2.2
> 6 months	263	97.8
Index wart previous treatment		
Yes	247	91.8
No	22	8.2

**Figure 2:**
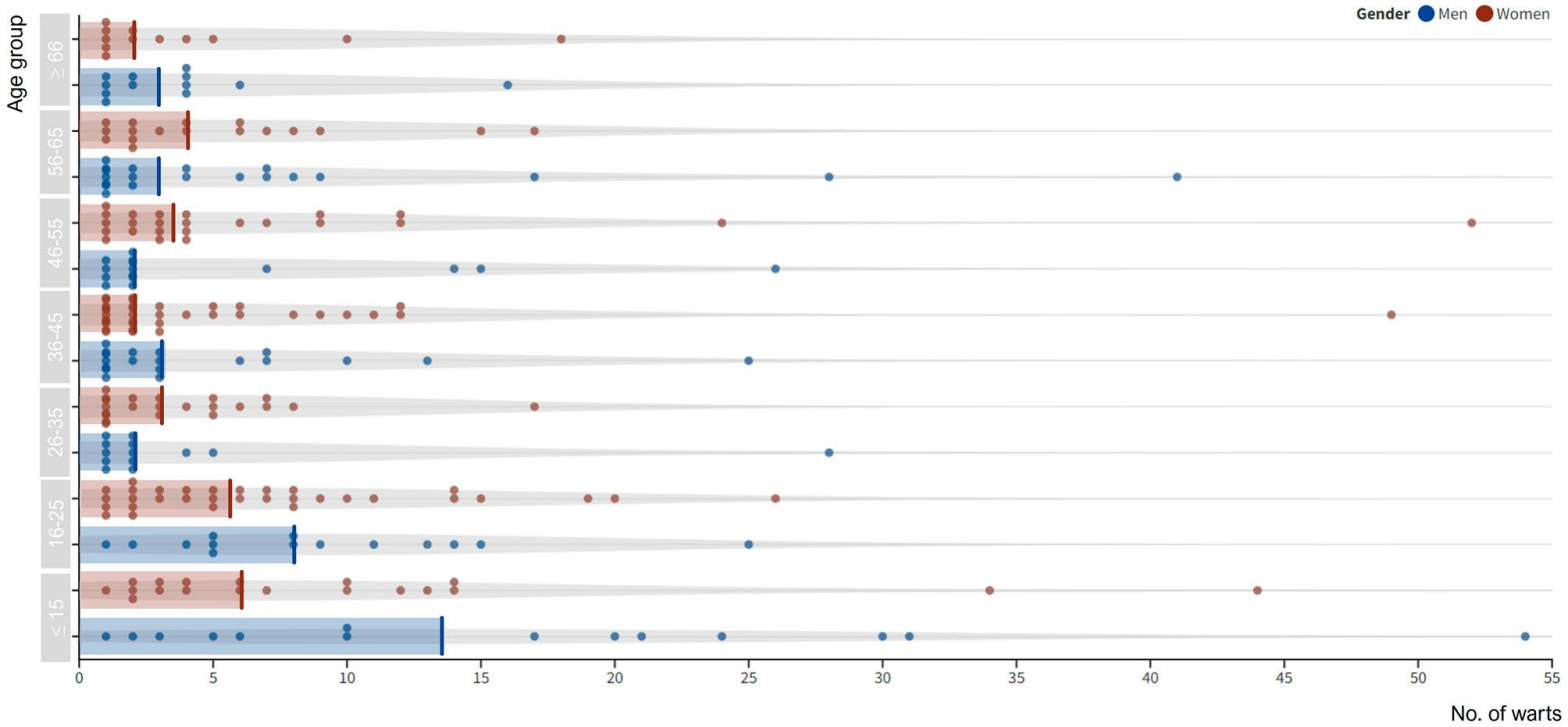
Number of warts per age group in the male and female population. Each study participant is depicted by a colored dot. Male patients are shown in blue, female patients in red. The number of patients per age group was respectively: 33 [≤ 15], 46 [16–25], 35 [26–35], 55 [36–45], 39 [46–55], 37 [56–65], and 24 [≥ 66]. The median number of warts per each age group is depicted by a colored line, with the highest median number of warts (13.5) found among patients aged ≤ 15 years. The highest number of warts per patient was found in age group ≤ 15 years and equaled to 54 warts in total.

All subjects are represented in Fig. 2 by the number of warts at the time of enrollment per age group and gender. There was no significant difference in the number of warts between the two genders (Student T-test *P* > 0.05). However, a significantly larger number of warts was found in the age groups ≤ 15 and 16–25 (Kruskal Wallis *P* < 0.005), suggesting that adolescents and young adults on average, exhibit a higher number of warts. The highest median number of warts was detected in male patients ≤ 15 years old (i.e. 13.5 warts per subject). No significant difference in the number of HPV multiple infections between different age groups was found (Kruskal–Wallis *P* > 0.05).

Considering the location of index warts, 85 (31.6%) were located on the hands, while 176 (65.4%) were located on the feet. Regarding index wart type, 89 (33.1%) of the index warts were verruca vulgaris, 113 (42.0%) were verruca plantaris mosaic, 60 (22.3%) were verruca plantaris simple, 6 (2.2%) were verruca plana and 1 (0.4%) was verruca filiformis. The average index wart size was 9 mm. Data showed that 263 (97.8%) of the index warts were older than 6 months and 247 (91.8%) were already subjected to prior treatment, indicating enrichment of the study population with highly persistent warts. *In casu*, 154 (62.3%) patients tried previous treatment with salicylic acid, 201 (81.4%) with cryotherapy, and 119 (48.2%) resorted to other treatments (i.e. treatment with other acids, bleomycin injections, fluorouracil creams, chirurgical and laser removal, and more systemic approaches based on cimetidine, zinc or magnesium). Multiple treatments were registered for 166 (61.7%) patients with two or more different treatment types (Fig. 1B).

### HPV type-specific prevalence

**Figure 3:**
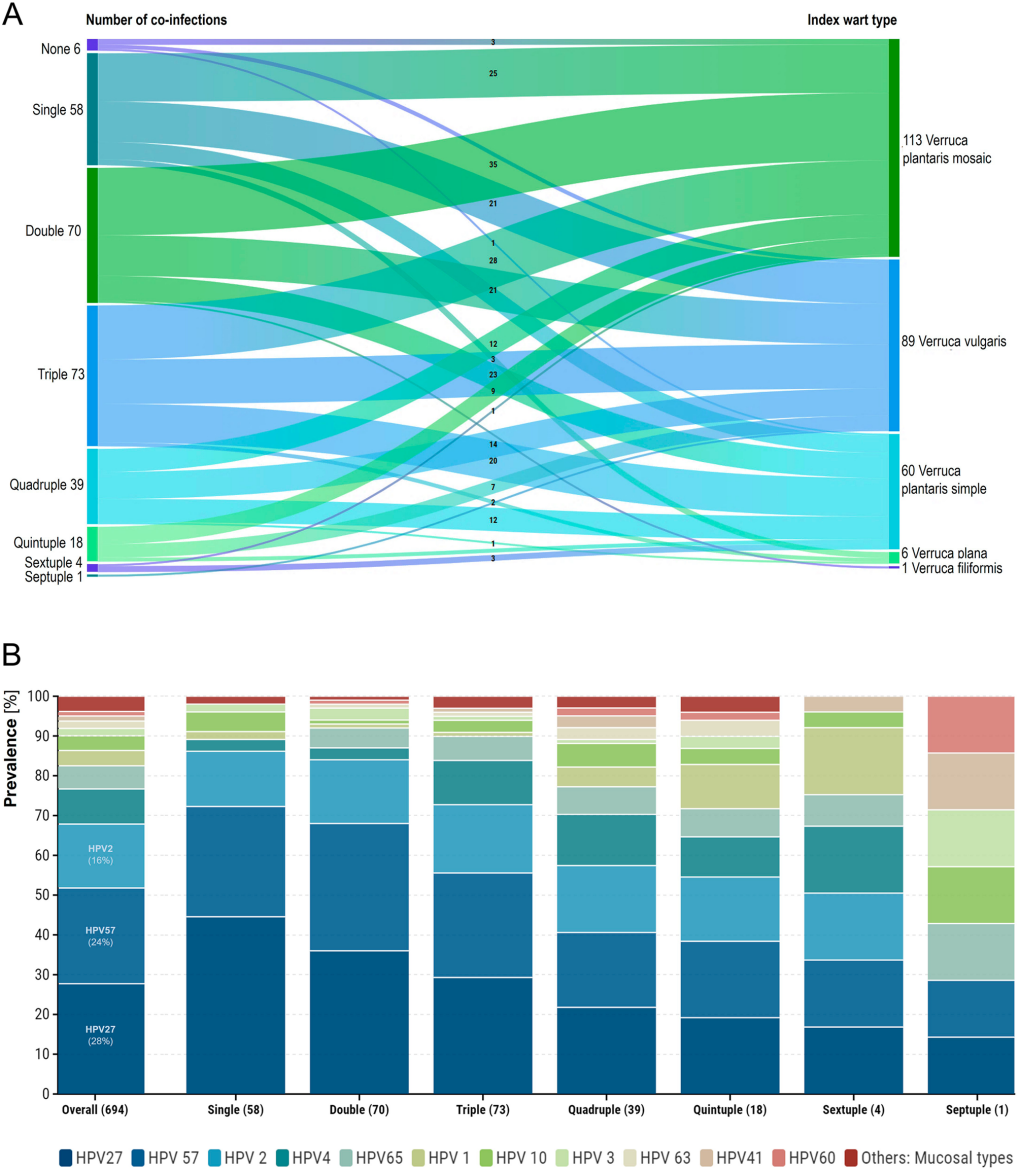
(**A**) Distribution of subjects based on their multiple infection status per specific index wart type. In summary, 25/58 (43%) of warts infected with a single HPV type were verruca plantaris mosaic, 21/58 (36%) verruca vulgaris, 9/58 (16%) verruca plantaris simple, 3/58 (5%) verruca plana. 35/70 (50%) of warts with double infections were verruca plantaris mosaic, 21/70 (30%) verruca vulgaris, 13/70 (19%) verruca plantaris simple, and 1/70 (1%) verruca filiformis. As regards to warts containing more than two distinct HPV types 50/135 (37%) were verruca plantaris mosaic, 45/135 (33%) verruca vulgaris, 37/135 (27%) verruca plantaris simple, and 3/135 (2%) verruca plana. (**B**) HPV type-specific prevalence in cutaneous warts. A total of 694 HPV infections was detected in the study population. Cutaneous HPV types 27 (28%, 195/694), 57 (24%, 169/694), and 2 (16%, 113/694) were the most commonly found types, with HPV type 7 being the exception, as it was not detected in this population. Certain mucosal HPV types (i.e. HPV 6, 31, 39, 51, 52, 53, 59, 66 and 68) were detected in low percentages (< 2%) and are depicted in group (red). The HPV type-specific distribution according to number of multiple infections is displayed as well with the number of subjects in each group between brackets.

As regards to sample quality, 261 (97.0%) samples were considered valid as demonstrated by a sufficient B-globin amplification. Moreover, patients showed a high HPV positivity rate where only 6 (2.2%) lesions were HPV negative, while 58 (21.6%) were positive for a single HPV genotype, and 205 (76.2%) contained multiple HPV infections. A high level of multiple infections was observed in 135 (50.2%) cases harboring three or more HPV infections. The maximum number of multiple infections in one patient was seven. Figure 3A provides an illustration off all subjects based on number of multiple infections per specific index wart type. The HPV multiple infection status is not dependent on wart type, seeing that similar distributions of number of infections can be found in all wart types identified. The most prevalent HPV types were cutaneous HPV types 27 (195/269; 72.5%), 57 (169/269; 62.5%) and 2 (113/269; 42.0%) (Fig. 3B). Only 2% (5/263) of HPV-positive warts were negative for HPV 2, 27 or 57. HPV 65 and 4 were present in 15–23% of samples, while HPV 1 and 10 in 10%, and HPV 3, 41, 60 and 63 in 3–5%. One cutaneous HPV type was not found in our population i.e. HPV 7. In general of all the cutaneous types, HPV 7 has the lowest reported prevalence (0.5%) and is often associated with meat handlers^27,41^. Mucosal HPV types were detected in 7% of lesions (18/269), more specifically high-risk HPV (HR-HPV) types 31, 39, 51, 52, 59, 66, possible HR-HPV 67, and low-risk HPV (LR-HPV) type 6 with respective prevalence ranging from 0.4–1.9% (Fig. 3B). Mucosal HPV types 16 and 18, most commonly associated with cervical cancer, were not found in this population (for more details on HPV type-specific prevalence see Additional file 1).

A full overview of genotype-specific prevalence can be found in Fig. 3B. Overall 26 (45%) warts were HPV 27 positive, 16 (28%) HPV 57, and 8 (14%) HPV 2. Other HPV types found in single infections were HPV 1, 3, 4, 10 and 31, with prevalence ranging from 2–5%. As outlined above, most warts displayed infection with multiple HPV types. Most common coinfections detected included HPV 27 and 57, and were observed in combination with at least one of the HPV types belonging to the alpha 4 (i.e. HPV 2, 27, 57), gamma 1 (i.e. HPV 4, 65) and/or gamma 4 species (i.e. HPV 60; Table 2)^20^.

**Table 2:** Most prevalent multiple infection trends in HPV-positive index warts.

Prevalent multiple infection trends	Samples (n = 263)	Percentage in population (%)
Double infection		
HPV 2 and 27	11	4.1
HPV 2 and 57	8	3.0
HPV 27 and 57	32	11.9
Triple infection		
HPV 2, 27 and 57	25	9.3
HPV 27, 4 and 57	8	3.0
HPV 27, 4 and 65	5	1.9
Quadruple infection		
HPV 2, 4, 27 and 57	8	3.0

Viral loads ranged from 1.11E−03 to 9.31E+06 copies per cell and showed a type-specific manifestation (Table 3). As for cutaneous HPV types, highest median viral loads were observed for HPV 27 (6.29E+04 copies per cell) and 57 (7.47E+01 copies per cell), and these differed significantly from viral loads of other types (Kruskal–Wallis *P* < 0.000001). Other genotypes displayed median viral loads ranging from 6.61E−02 to 2.39E+00 copies per cell for cutaneous types, and from 1.98E+01 to 6.09E+04 for mucosal types. The type-specific viral loads found in single infections did not significantly differ from those found in multiple infections within the same HPV type (Student T-test *P* > 0.05).

**Table 3:** Viral load quantification of identified HPV types.

HPV type	n	Median viral load copy/cell (IQR)
Cutaneous		
HPV 1	27	1.87E−01 (5.16E−02–1.08E+00)
HPV 2	112	2.39E+00 (2.43 E−01–4.64E+04)
HPV 3	12	1.21E−01 (3.59E−02–2.75E−01)
HPV 4	60	6.61E−02 (2.36E−02–7.63E−01)
HPV 7	–	NA
HPV 10	26	7.18E−01 (8.81E−02–3.28E+00)
HPV 27	193	6.29E+04 (7.40E+00–2.05E+05)
HPV 41	9	4.47E+01 (9.48E−02–3.18E+00)
HPV 57	167	7.47E+01 (3.50E+00–9.68E+04)
HPV 60	8	1.10E+00 (2.83E−01–3.20E+00)
HPV 63	12	1.11E−01 (6.09E−02–7.05E−01)
HPV 65	45	2.85E+00 (5.42E−01–2.96E+01)
Mucosal		
HPV 6	1	6.09E+04 (NA)
HPV39	2	3.66E+04 (1.86E+04–5.46E+04)
HPV51	4	1.55E+02 (4.79E+01–3.54E+02)
HPV52	1	1.47E+03 (NA)
HPV53	5	4.51E+01 (3.79E+01–5.20E+01)
HPV59	2	5.14E+01 (3.13E+01–7.14E+01)
HPV66	1	1.98E+01 (NA)
HPV67	1	3.69E+04 (NA)

The table depicts the number of index warts (n) positive for each HPV type and the median viral load per specific type. Viral load is expressed as number of viral copies per cell. Viral load inter quartile range (IQR) per type is likewise described.

### Predictors of wart persistence

After adjusting for all other covariates in the multivariate model, there were four significant associations with the persistence of the index wart (Table 4). Compared to the verruca plantaris mosaic, warts from the “others” category had 86% (95% CI 0.03–0.83) lesser odds of persistence, although the sample size was small (n = 7). Furthermore, as predicted, having two or more warts was significantly associated with the outcome, with patients harboring two or more warts having 2.65 higher odds (95% CI 1.07–6.55) of persistence than patients with one wart. Concerning HPV genotypes, only HPV 2, and HPV 4 were found to be statistically associated with the outcome, with their presence resulting in a respective decrease of 66% and 70% odds of index wart persistence.

**Table 4:** Association of several covariates with wart persistence: multivariable logistic regression analysis.

Variable	Unadjusted odds ratio (95%CI)	*P*-value	Adjusted odds ratio (95%CI)	*P*-value
Index wart type				
Verruca plantaris mosaic	Baseline			
Verruca vulgaris	0.50 (0.17–1.46)	0.203	0.75 (0.26–2.20)	0.606
Verruca plantaris simple	0.28 (0.10–0.81)	0.019	0.49 (0.16–1.50)	0.211
Other	0.14 (0.02–0.88)	0.036	0.14 (0.03–0.83)	0.030
Number of warts				
1	Baseline			
≥ 2	2.34 (1.02–5.33)	0.044	2.65 (1.07–6.55)	0.035
Number of infections				
0	Baseline			
1	2.12(0.21–21.89)	0.530	3.11 (0.36–26.87)	0.302
> 2	1.75(0.19–15.72)	0.612	3.93 (0.31–50.52)	0.293
Cutaneous HPV type				
HPV 1	0.60 (0.19–1.90)	0.390	0.62 (0.18–2.17)	0.459
HPV 2	0.38 (0.17–0.88)	0.024	0.34 (0.12–0.99)	0.048
HPV 3	1.36 (0.17–10.86)	0.770	0.66 (0.08–5.07)	0.693
HPV 4	0.33 (0.14–0.74)	0.007	0.30 (0.11–0.79)	0.015
HPV 10	1.38(0.32–6.12)	0.680	1.61 (0.35–7.46)	0.541
HPV 27	1.36 (0.58–3.18)	0.480	1.36 (0.48–3.85)	0.562
HPV 57	1.40 (0.63–3.13)	0.410	1.26 (0.47–3.35)	0.644
HPV 63	3.20 (0.19–55.94)	0.420	2.20 (0.12–42.03)	0.600
HPV 65	1.04 (0.34–3.18)	0.940	1.30 (0.38–4.51)	0.676

## Discussion

In this study, a vast prevalence of HPV was found in cutaneous warts, with only a limited number of warts identified as negative for the analyzed HPV genotypes (2%). From a pre-analytical perspective, our findings confirm the efficient collection of test samples via a non-invasive, swab-based technique. The efficiency of painless collection via surface swabs has been described rendering swab samples a reliable tool to test for viral presence in skin lesions^32,42,43^. It can be expected that by circumventing the need for invasive sample collection, the potential of HPV genotyping in cutaneous lesions will be more easily exploited as patient’s reluctance to undergo testing will be substantially reduced as well as the need for highly experienced professionals to apply the technique^43^.

In concordance, the observed high HPV positivity rate also confirms efficiency at the analytical level. The cutaneous wart-associated HPV genotyping assay was carefully designed to ensure maximum sensitivity^33^, simultaneously allowing viral load determination. Due to this high sensitivity, HPV types were detected with high accuracy, resulting in the identification of at least one cutaneous HPV type in 97% of all samples, with HPV 27 (73%), 57 (63%) and 2 (42%) being the most prevalent types. This high cutaneous HPV type-specific prevalence further confirms that the most relevant HPV genotypes were selected and included in the cutaneous wart-associated HPV assay. Likewise, the RIATOL qPCR HPV assay, capable of detecting mucosal HPV types, has been exploited at full analytical potential without the use of the previously described clinical cut-off for primary cervical cancer^35^. This assay is highly sensitive and allows viral load determination even at low cell counts. The high sensitivity of the employed assays correspondingly explains the high prevalence of multiple infections (76.2%), which is in contrast with current literature reporting only 4–46% multiple infections in immunocompetent subjects using similar sampling techniques^27,42,44,45^. However, Schmitt et al. did report the highest prevalence of multiple infections in swab samples currently published (46%), and confirmed that use of a more sensitive method leads to a superior ability to detect multiple HPV infections in the same swab sample.

Thus far, the largest study regarding HPV prevalence in cutaneous warts was conducted by Bruggink et al. and comprised 744 warts of 246 immunocompetent patients^27^. The lower prevalence of multiple infections found, can be explained by the fact that in their study individual warts are considered as the unit of analysis instead of patients i.e. multiple warts of the same patients were sampled for further HPV analysis, propagating therefore repetitive results in the population. Furthermore, exclusively ‘new’ warts were included in their study, defined as warts without any prior treatment from a general practitioner or dermatologist^27^. Considering that in the current study, 92% of subjects were already subjected to prior treatment, the high percentage of multiple infections could be an indicator of persistence. In addition, Bruggink et al. excluded mosaic warts with a diameter of ≥ 1 cm from their study, while this type of warts compromised 20% (n = 55) of our population^27^.

To the best of our knowledge, the most comprehensive study exploring HPV genotyping and including a viral load component was the study conducted by Skubic et al.^46^. In contrast, the latter study focused on histological specimens, hereby generating strong evidence on genotype distribution and investigating the role of HPV viral load in cutaneous warts^46^. Our findings, obtained by the collection of cutaneous swabs, largely confirm the findings by Skubic et al., showing the highest prevalence of HPV 27 and 57, with comparable levels of viral load distribution as well, despite essentially different starting material. Based on observations by de Koning et al. as well as Garcia-oreja et al., high concordance between cutaneous swabs and biopsies can be expected, thus strengthening findings from this study^42,43^.

In this population, HPV 27 and 57 exhibited significantly higher viral loads in comparison to other types, correlating with high viral shedding and associated highest infectious potential, which is translated in highest prevalence in the study population^42,47,48^.

The multivariate analysis found significant associations between wart persistence and certain wart types, number of warts and HPV genotypes. Having verruca plana or filiformis significantly decreased the risk of persistence in comparison to verruca plantaris mosaic. The mosaic wart is already recognized as the most persistent wart type and was as well enriched in this study population (42%)^37^. Number of warts being a risk factor for persistence is to be expected, knowing that persistent warts are resistant to treatment and, by definition, have a duration longer than 6 months, which increases the risk of autoinoculation and transmission of the HPV infection from one body site to another. As regards to the role of HPV genotyping, seeing that the high viral load of HPV 27 and 57 results in a subsequent high infectious potential, these types appear to be omnipresent in the population, not only in persistent warts but also in warts that are defined as not persistent^3,27,46^. This finding makes them an inadequate predictor of risk. However, the logistical regression did show that some HPV types are in fact, less likely to cause persistent warts i.e. HPV 2 and 4 (OR 0.38 and 0.31 respectively). HPV 4 has previously been identified by Bruggink et al. as having the most favorable natural course in plantar warts (cure rate 94% (95% CI 73–99%))^25^. HPV 2, however, did not have a promising natural course in their analysis (cure rate 0% (95% CI 0–26) in plantar warts and 3% (95% CI 0–16) in common warts). Claims about cure rates of other HPV types were also not possible, due to a lack of sufficient numbers per HPV type in different treatment groups. Despite certain discrepancies, our findings together with Bruggink et al. do consolidate the future applicability of HPV genotyping in clinical management of cutaneous warts.

## Conclusions

Given the high prevalence of HPV types in cutaneous lesions, and their relationship with the clinical manifestation of warts, HPV genotyping with viral load determination can be of added value in daily clinical practice. Combined with the knowledge that the persistence of a lesion is linked to both the HPV genotype, wart type and number of warts, risk assessment can be performed, and therapeutical options can be suggested. In case that the above-defined risk factors are present in a subject, a more aggressive treatment approach can be applied in order to inhibit not only the spread of the infection in the general population but also further autoinoculation. Previous studies are in line with these findings. However, further research is needed to compose a comprehensive tool to guide clinicians in wart treatment and/or follow-up. For a more extensive assessment of HPV-dependent wart persistence, upcoming studies must entail large clinical trials comprising not only different treatment groups (e.g. salicylic acid, cryotherapy and wait-and-see) but also ensuring a sufficient HPV type-specific allocation in each group. Only then, a head-to-head comparison of different genotypes is possible, creating the possibility to make distinct claims about treatment response and natural course based on HPV type. Nevertheless, the present study already provides certain insights in possible predictors of wart persistence and encourages a more patient-centered and directed approach to wart risk and treatment stratification.

### Supplementary Information

Supplementary Information.

## Data Availability

The data that support the findings of this study are available from the corresponding author upon reasonable request.

## Abbreviations

AIC: Akaike information criterion
BIC: Bayesian Information Criterion
HPV: Human Papilloma Virus
HR-HPV: High-Risk Human Papilloma Virus
IQR: Inter Quartile Range
LR-HPV: Low-Risk Human Papilloma Virus
OVW-SA: Omnivirol-Salicylic acid combination therapy for cutaneous warts
qPCR: Real-Time Polymerase Chain Reaction
VIF: Variance Inflation Factor

## References

[1] Hashmi F (2015). EVerT2-needling versus non-surgical debridement for the treatment of verrucae: Study protocol for a singlecentre randomised controlled trial. BMJ Open.

[2] Sterling JC, Gibbs S, Haque HSS, Mohd MMF, Handfield-Jones SE (2014). British Association of Dermatologists’ guidelines for the management of cutaneous warts 2014. Br. J. Dermatol..

[3] Breznik V (2020). Determination of causative human papillomavirus type in tissue specimens of common warts based on estimated viral loads. Front. Cell Infect. Microbiol..

[4] Cardoso JC, Calonje E (2011). Cutaneous manifestations of human papillomaviruses: A review. Acta Dermatovenerol Alp Pannonica Adriat..

[5] Lipke MM (2006). An armamentarium of wart treatments. Clin. Med. Res..

[6] Surentheran T (1998). Detection and typing of human papillomaviruses in mucosal and cutaneous biopsies from immunosuppressed and immunocompetent patients and patients with epidermodysplasia verruciformis: A unified diagnostic approach. J. Clin. Pathol..

[7] Porro AM (2003). Detection and typing of human papillomavirus in cutaneous warts of patients infected with human immunodeficiency virus type 1. Br. J. Dermatol..

[8] De Koning MNC (2015). High prevalence of cutaneous warts in elementary school children and the ubiquitous presence of wart-associated human papillomavirus on clinically normal skin. Br. J. Dermatol..

[9] Massing A, Epstein W (1963). Natural history of warts. A two year study. Arch. Dermatol..

[10] Lynch MD, Cliffe J, Morris-Jones R (2014). Management of cutaneous viral warts. BMJ.

[11] Journal B (2001). Guidelines for the management of cutaneous warts. Br. J. Dermatol..

[12] Micali G, Dall’Oglio F, Nasca MR (2013). Topical treatments for cutaneous warts: An update. Expert Rev. Dermatol..

[13] Kollipara R (2015). Advancements in pharmacotherapy for noncancerous manifestations of HPV. J. Clin. Med..

[14] Bacelieri R, Johnson SM (2005). Cutaneous warts: An evidence-based approach to therapy. Science.

[15] Gearhart P, Randall T, Buckley RM, Higgins R (2016). Human papillomavirus treatment and management. Medscape.

[16] Gibbs S, Harvey I, Gibbs S (2006). Topical treatments for cutaneous warts. Cochrane Database of Systematic Reviews.

[17] Gibbs S, Harvey I (2006). Topical treatments for cutaneous warts (Review). Cochrane Database Syst. Rev..

[18] Kuwabara AM, Rainer BM, Basdag H, Cohen BA (2015). Children with warts: A retrospective study in an outpatient setting. Pediatr. Dermatol..

[19] Burd EM (2003). Human papillomavirus and cervical cancer. Clin. Microbiol. Rev..

[20] De Villiers EM, Fauquet C, Broker TR, Bernard HU, Zur Hausen H (2004). Classification of papillomaviruses. Virology.

[21] Bzhalava D, Guan P, Franceschi S, Dillner J, Clifford G (2013). A systematic review of the prevalence of mucosal and cutaneous human papillomavirus types. Virology.

[22] Giannaki M (2013). Human papillomavirus (HPV) genotyping of cutaneous warts in greek children. Pediatr. Dermatol..

[23] Michael KM (2011). Bead-based multiplex genotyping of 58 cutaneous human papillomavirus types. J. Clin. Microbiol..

[24] De Koning MNC (2010). Evaluation of a novel broad-spectrum PCR-multiplex genotyping assay for identification of cutaneous wart-associated human papillomavirus types. J. Clin. Microbiol..

[25] Bruggink SC (2013). HPV type in plantar warts influences natural course and treatment response: Secondary analysis of a randomised controlled trial. J. Clin. Virol..

[26] Hogendoorn GK (2018). Morphological characteristics and human papillomavirus genotype predict the treatment response in cutaneous warts. Br. J. Dermatol..

[27] Bruggink SC (2012). Cutaneous wart-associated HPV types: Prevalence and relation with patient characteristics. J. Clin. Virol..

[28] Redzic N, Benoy I, Vanden BD, Bogers JP (2020). Efficacy of AV2-Salicylic acid combination therapy for cutaneous warts: Study protocol for a single-center randomized controlled trial. Contemp. Clin. Trials Commun..

[29] Redzic N, Benoy I, Vanden BD, Bogers JP (2020). Efficacy of AV2-Salicylic acid combination therapy for cutaneous warts: Study protocol for a single-center randomized controlled trial. Contemp. Clin. Trials Commun..

[30] Directive 05/28/EC of the European Parliament and of the Council of of 8 April 2005 laying down principles and detailed guidelines for good clinical practice as regards investigational medicinal products for human use, as well as the requirements for authorisation of the manufacturing or importation of such products. *Official J. Eur. Union* 13–19 (2005).

[31] World Medical Association Declaration of Helsinki (2013). JAMA.

[32] Redzic N, Pereira AR, Vanden BD, Bogers JP (2023). Optimizing the pre-analytical phase for accurate HPV detection in skin disorders: Insights from a cutaneous warts case study. Ther. Adv. Infect. Dis..

[33] Redzic N, Benoy I, Vanden Broeck D, Bogers JP (2021). Development and validation of a wart-associated human papilloma virus genotyping assay for detection of HPV in cutaneous warts. J. Med. Virol..

[34] Micalessi IM, Boulet GAV, Bogers JJ, Benoy IH, Depuydt CE (2012). High-throughput detection, genotyping and quantification of the human papillomavirus using real-time PCR. Clin. Chem. Lab Med..

[35] Benoy I (2019). Using the VALGENT-3 framework to assess the clinical and analytical performance of the RIATOL qPCR HPV genotyping assay. J. Clin. Virol..

[36] Depuydt CE (2012). Clinical validation of a type-specific real-time quantitative human papillomavirus PCR against the performance of hybrid capture 2 for the purpose of cervical cancer screening. J. Clin. Microbiol..

[37] Cockayne S (2011). Cryotherapy versus salicylic acid for the treatment of plantar warts (verrucae): A randomised controlled trial. BMJ.

[38] Bruggink SC (2013). Natural course of cutaneous warts among primary schoolchildren: A prospective cohort study. Annals Family Med..

[39] Johnston R, Jones K, Manley D (2018). Confounding and collinearity in regression analysis: A cautionary tale and an alternative procedure, illustrated by studies of British voting behaviour. Qual. Quant..

[40] Collins LM, Schafer JL, Kam C-M (2001). A comparison of inclusive and restrictive strategies in modern missing data procedures. Psychol. Methods.

[41] Villiers E-M, De Neumann C, Oltersdorf T, Fierlbeck G, Hausen HZ (1986). Butcher’s Wart Virus (HPV 7) infections in non-butchers. J. Invest. Dermatol..

[42] de Koning MNC (2011). Lesional HPV types of cutaneous warts can be reliably identified by surface swabs. J. Clin. Virol..

[43] García-Oreja S (2022). A non-invasive method for diagnosing plantar warts caused by human papillomavirus (HPV). J. Med. Virol..

[44] Schmitt M, De Koning MNC, Eekhof JAH, Quint WGV, Pawlita M (2011). Evaluation of a novel multiplex Human Papillomavirus (HPV) genotyping assay for HPV types in skin warts. J. Clin. Microbiol..

[45] Vlahovic TC, Khan MT (2016). The human papillomavirus and its role in plantar warts. Clin. Podiatr. Med. Surg..

[46] Skubic L (2022). An improved protocol for comprehensive etiological characterization of skin warts and determining causative human papillomavirus types in 128 histologically confirmed common warts. Viruses.

[47] Lei Y-J (2009). Molecular epidemiological study on prevalence of human papillomaviruses in patients with common warts in Beijing area. Biomed. Environ. Sci..

[48] Köhler A, Meyer T, Stockfleth E, Nindl I (2009). High viral load of human wart-associated papillomaviruses (PV) but not β-PV in cutaneous warts independent of immunosuppression. Br. J. Dermatol..

