# Supplementary material for: Characterization of type-specific HPV prevalence in a population of persistent cutaneous warts in Flanders, Belgium
Source: Sci Rep. 2023 Oct 15;13:17492. doi: 10.1038/s41598-023-44154-y (PMC10577142; doi:10.1038/s41598-023-44154-y)
Supplement: Supplementary file 1 — Supplementary Information. [file 41598_2023_44154_MOESM1_ESM.docx]

**Additional file 1**

| **Table 1:** Details of outcome measures and data collection forms used in the OVW-SA001 clinical trial. The majority of the outcomes are responses to questions that require a ‘Yes’ or ‘No’ answer or open comments, except for pain which was measured by a 0-4 numeric pain rating scale. | |
| --- | --- |
| **Measure** | **Source: Content** |
| Eligibility | **Inclusion Criteria**:  patient exhibiting one or more cutaneous warts; aged 12 years or older; agrees to refrain from using prescription or supplemental antiviral medications without first obtaining permission of the coordinating trial dermatologist; able to read Dutch; signed informed consent; able to self-assess and use WhatsApp for follow-up (all Y/N).  **Exclusion Criteria**:  patient only exhibiting facial and/or seborrheic warts; not suitable for salicylic acid (SA) treatment due to a medical history of severe diseases (e.g. hepatitis, renal or liver dysfunction, cardiovascular, or gastrointestinal disorders, etc.), impaired healing or neuropathy (e.g. due to diabetes, peripheral vascular disease or any other condition); known or suspected allergic or adverse response to SA, AV2 or its components; immunocompromised patient; patient had already participated in another clinical trial concerning treatment for cutaneous warts within six months before enrolment in this study or is currently in a trial evaluating other treatments for his/her warts (all Y/N). |
| Demographic  Details | **Baseline Questionnaire**:  date of birth; sex (M/F); postal code. |
| Wart  anamnesis | **Baseline Questionnaire**:  General History:  number of warts; wart type (verruca vulgaris, verruca plantaris mosaic or simple, verruca plana, verruca filiformis); average size (mm); location; duration (<6months, >6months); previous treatment (Y/N, if yes specify).  Index Wart History:  wart type; average size (mm); location; duration (<6months, >6months). |
| Efficacy Of  Treatment | **Follow-Up Questionnaire:**  Index wart:  clearance (Y/N) → if cleared date of clearance; if not cleared potential recurrence Y/N.  Other warts:  still present (Y/N) → if not present date of clearance; if present number of warts and location (inside a radius of 0.5cm around the original position/another position = ‘new warts’ → if new warts, inside a radius of 3cm around the index lesion (Y/N; if yes number of warts). |
| Side Effects Of  Treatment | **Follow-Up Questionnaire:**  pain scores (numeric pain rating scale 0=no pain at all – 4=extreme level of pain); another side effects (Y/N, if yes specify). |
| Treatment  Compliance | **Follow-Up Questionnaire:**  use of additional treatments (Y/N; if yes specify); comments about treatment (open text). |
| Photograph-  Based Outcome  Assessment | **Photograph-Based Assessment Form:**  Study Coordinator:  date at which the photograph is taken; treatment day; index wart cleared (Y/N); size of index wart (mm).  Dedicated Physician:  photograph interpretable (Y/N); index wart cleared (Y/N); remarks (open text). |
| HPV  Genotyping | **Laboratory Form:**  date at which the sample is taken; treatment day; HPV (pos/neg; if pos specify HPV type(s)). |


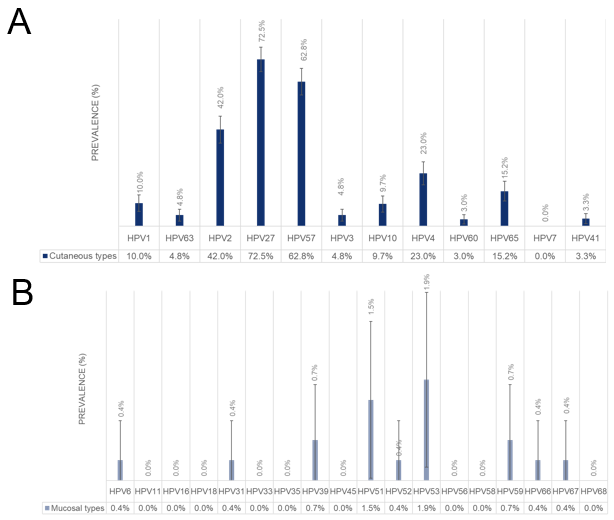


**Figure 1.** HPV type-specific prevalence in cutaneous warts with 95% Confidence Intervals: **(A)** cutaneous HPV types and **(B)** mucosal HPV types.
